# Revisiting the Concepts of Prebiotic and Prebiotic Effect in Light of Scientific and Regulatory Progress—A Consensus Paper From the Global Prebiotic Association

**DOI:** 10.1016/j.advnut.2024.100329

**Published:** 2024-10-29

**Authors:** Edward C Deehan, Santa Al Antwan, Rhonda S Witwer, Paula Guerra, Tania John, Len Monheit

**Affiliations:** 1 Department of Food Science and Technology, University of Nebraska, Lincoln, NE, United States; 2 Nebraska Food for Health Center, University of Nebraska, Lincoln, NE, United States; 3 Scientific & Technical Committee, Global Prebiotic Association, Chicago, IL, United States; 4 SGS Nutrasource, Guelph, Ontario, Canada; 5 ADM, Decatur, IL, United States; 6 Global Prebiotic Association/Industry Transparency Center, Chicago, IL, United States

**Keywords:** prebiotic, prebiotic effect, definition, gut microbiome, health benefits

## Abstract

The term prebiotic has been used for almost 3 decades and has undergone numerous updates over the years. The scientific literature reveals that despite continuous efforts to establish a globally unified definition to guide jurisdictional regulations and product innovations, ambiguity continues to surround the terms prebiotic and prebiotic effect, leading to products that lack in full regulatory adherence being marketed worldwide. Thus, to reflect the current state of scientific research and knowledge and for the continuous advancement of the category, an update to the current prebiotic definition is warranted. This update includes removing the term selectivity, considering additional locations of action besides the gut, highlighting prebiotic performance benefits such as cognitive and athletic, and providing a clear standalone definition for prebiotic effect. The Global Prebiotic Association (GPA) is a leading information and industry hub committed to raising awareness about prebiotics, their emerging and well-established health benefits, and prebiotic product integrity and efficacy. In this position paper, GPA builds on previous prebiotic definitions to propose the following expanded definition for prebiotic: “a compound or ingredient that is utilized by the microbiota producing a health or performance benefit.” In addition to prebiotic, GPA also defines prebiotic effect as “a health or performance benefit that arises from alteration of the composition and/or activity of the microbiota, as a direct or indirect result of the utilization of a specific and well-defined compound or ingredient by microorganisms.” With these 2 definitions, GPA aims to paint a clearer picture for the term prebiotic, and by incorporating an industry point of view, these updated definitions may be used alongside current scientific and regulatory perspectives to move the category forward.

**Statement of Significance**

The purpose of this article is to revisit the concepts of prebiotic and prebiotic effect by providing a scientific-based industry perspective. The proposed definitions of prebiotic and prebiotic effect reflect recent discoveries in metagenomics and prebiotic research since the International Scientific Association for Probiotics and Prebiotics 2017 prebiotic definition and propose terminology changes that are timely and necessary. These changes aim to maintain the clarity and usefulness of the prebiotic definition to the scientific community, industry, healthcare providers, and consumers, while ensuring scientific validity, comprehensiveness, and justification of each part of the prebiotic definition, including abandoning the term selectivity and introducing concepts of performance benefits and prebiotic effect.

## Introduction

A myriad of bacteria, viruses, fungi, archaea, and protozoans reside in human body, with over a 1:1 ratio to human cells in an adult human, totaling roughly 39 trillion microbes with taxonomically complex and ecologically dynamic natures [1,2]. These microbes are found on the skin and in the respiratory system, urogenital tract, and gastrointestinal (GI) tract as their primary neighborhood, with an extremely dense ecosystem centered within the colon [[3], [4], [5]]. The assembly of these microorganisms in a defined environment is known as the microbiota, while the cells with their collective genomes and the surrounding environmental conditions form the microbiome [6,7]. Thus, the primary aims of microbiome research are *1*) taxonomic diversity to discover the microbiota or residing strains, and *2*) functional metagenomics to identify their roles within the environment [4].

By numbers, over 70% of the microbiome resides in the GI tract and consists of ∼400–1500 different species, with varying composition across individuals [8,9]. The dominant gut-colonizing microbes are bacterial, including the phylum Bacillota (previously Firmicutes), which encompasses *Lactobacillus*, *Bacillus*, *Clostridium*, *Enterococcus*, *Ruminococcus*, *Eubacterium*, *Faecalibacterium*, and *Roseburia* spp. The other dominant phylum in the gut is Bacteroidota (previously Bacteroidetes), which encompasses *Bacteroides* and *Prevotella* spp. Other phyla include Actinomycetota (previously Actinobacteria) (e.g. *Bifidobacterium* sp.), Pseudomonadota (previously Proteobacteria), and Verrucomicrobiota (previously Verrucomicrobia) (e.g. *Akkermansia* sp.) [10]. The gut microbiome also comprises pathobionts—organisms native to the host but can cause harm under select conditions—such as *Escherichia coli*, *Clostridioides difficile*, and *Enterococcus* sp.[11]. However, a clear distinction between beneficial and harmful bacteria remains controversial as current evidence suggests that effects on health are context dependent (i.e. strain and dietary factors) [4,12]. Consequently, defining a healthy microbiome has been a challenge, as well as the development of effective microbiome-targeting strategies to promote health. In general, a healthy microbiome is characterized by high microbial diversity, and although relatively stable in adulthood, factors such as environmental disruptions (i.e. physical activity, mode of delivery, medication use, and diet) can influence microbiome diversity. While research to advance the global understanding of the gut microbiome and what constitutes a healthy microbiome is ongoing, it is generally agreed upon that additional factors such as the presence of select beneficial microbial species and their metabolic activities, relevant host–microbe interactions, and the functional capacity of the gut microbiome contribute to different biological and disease processes in humans [13,14].

Diet contributes to microbiome composition, and close relationships have been established among dietary changes, microbiome composition, and health [1]. Epidemiological data suggest that diet-induced changes to gut-associated bacteria have contributed to the growing noncommunicable disease epidemics in the developed world, including obesity [15], inflammatory bowel disease [16], metabolic disorders [17], and cancer [18]. Gut bacteria utilize diet-derived nondigestible components as their major energy source [3]; however, in their absence, some members can use host-derived components (e.g. mucin, the primary structural component of mucus) [19]. The intake of nondigestible dietary components can influence the gut bacterial community by altering microbial fermentation, bacterial community composition, and metabolite production in the large intestine [6,20]. Some of these dietary compounds, such as prebiotics, can positively influence the gut microbiome and human health [21,22].

The microbial fermentation of prebiotics can promote putatively beneficial bacteria within the gut and produce metabolites that further manipulate the bacterial community and its metabolic activity [23]. Thus, prebiotics influence gut microbiome composition [22,24,25], conferring an effect on microbiome-linked diseases. Evidence from clinical and preclinical studies have shown positive actions of prebiotics in different health conditions, including metabolic disorders [[26], [27], [28], [29]], those that are bone related [30] and GI related [31,32], and more recently, mental health [33,34]. With evidence accumulating, the scientific community found it necessary to define these substrates.

Historically, the first attempt to define prebiotics was by Gibson and Roberfroid in 1995 as “nondigestible food ingredients that beneficially affect the host by selectively stimulating the growth and/or activity of one or a limited number of bacterial species already resident in the colon, and thus attempt to improve host health.” In subsequent years, multiple research groups built upon the definition in an attempt to guide the growing prebiotic field, introducing revisions to the original terminology regarding classes of ingredients (i.e. nondigestible carbohydrates), mechanisms of action (i.e. selective utilization by the microbiota), and location of the affected microbial communities (i.e. gut) [24,[35], [36], [37], [38], [39], [40], [41]]. While the original term was coined to describe nondigestible or absorbable carbohydrate structures, other substances like polyunsaturated fatty acids and polyphenols have also shown prebiotic potential [23,42,43]. As a result, the International Scientific Association for Probiotics and Prebiotics (ISAPP) published an updated definition in 2017 that included these novel prebiotic types [i.e. recently identified prebiotic candidates with increasing research of their prebiotic efficacy such as polyphenols and ω-3 (n–3) fatty acids], defining a prebiotic as “a substrate that is selectively utilized by host microorganisms conferring a health benefit” [42]. Today, the debate on the definition of prebiotics continues while guidance on defining other relevant terms like prebiotic activity or effect remains absent.

As the science continues to progress, it is evident that the definition of prebiotics must evolve as well. In addition, a clear differentiation between prebiotic benefit and prebiotic action/activity is needed. On the global regulatory front, in 2008, the FAO of the United Nations proposed an update to the 1995 definition from Gibson and Roberfroid [39]. Multiple countries in the European Union (EU) and across the world (e.g. Canada, Brazil, and Italy) either reference or align with the rigorous scientific evidence requirement of the FAO’s definition in their prebiotic regulations. Other regulatory authorities rely on ISAPP’s or one of the older definitions, including the United States Food and Drug Administration (FDA), which recognizes the 1995 definition by Gibson and Roberfroid [44]. Globally, food and supplement companies find this disconnect in understanding what constitutes a prebiotic between jurisdictions concerning as it hinders category advancement and public understanding; therefore, requiring an industry perspective from these groups that interact directly with end users, including consumers.

The Global Prebiotic Association (GPA), comprising ingredient manufacturers, brand holders, retailers, and service companies from around the world, is committed to raising awareness of prebiotics and their emerging and distinct health benefits. GPA is an information and industry strategy hub, interacting with and providing resources to stakeholders, the medical community, consumers, academia, media, as well as government and regulatory agencies. Its membership is dedicated to maintaining prebiotic product integrity and efficacy and building long-term awareness through education initiatives such as communicating health benefits to consumers. With recent discoveries in gut biology and prebiotic research, GPA initiated and led discussions to revisit the prebiotic definition, and initial thoughts naturally progressed into a dedicated work stream.

With this position paper, GPA aims to provide an industry perspective on prebiotics as an evolving market to protect the category and the research that informs it, contributing to the outstanding foundational work previously done by other organizations in the field.

## Purpose of Paper

Given the rise in novel prebiotic types, including formulas with broad actions, mechanisms influencing various microbiome locations, and effects extending to different bodily systems, it is necessary to revisit and update the prebiotic definition. Moreover, it is imperative to have a clear and inclusive definition to move the category forward with the most current scientific knowledge. This article aims to expand on previous prebiotic definitions by providing an industry perspective based on the review of current scientific literature as well as relevant discussions between experts from the industry and the scientific community.

The new proposed definition retains concepts further substantiated since ISAPP’s consensus definition in 2017, such as the inclusion of noncarbohydrate ingredients (i.e. polyphenols and ω-3 fatty acids) and non-GI microbiomes (i.e. skin, respiratory, and urogenital). While this definition abandons the concept of selectivity, it more importantly introduces 2 new concepts of relevance: performance benefit and prebiotic effect, which are discussed later in detail.

## Methods

Made up of over 40 members worldwide, GPA is a nonprofit trade association of ingredient manufacturers, brand holders, retailers, and associate members with expertise in prebiotic scientific innovation, substantiation, regulation, and marketing. GPA’s objective is to champion the prebiotic category by increasing awareness and understanding of the science supporting well-known and newfound benefits of prebiotics.

Through GPA-led review of current scientific literature and continuous discussion between industry and academic experts, a new consensus prebiotic definition was developed that retains clarity and scientific validity, is understandable by all stakeholders, is useful to further the regulatory dialog, and is comprehensive in its usage and justification of each part of the definition. The prebiotic and prebiotic effect definitions put forth by GPA include new ingredients (e.g. noncarbohydrate prebiotics) and formulations (e.g. topical), distant microbial sites beyond the gut (e.g. skin and urogenital), refer to health and performance benefits (e.g. athletic performance and cognitive performance) [33,45,46], cover both humans and animals, exclude the term selectivity due to its ambiguity and irrelevance with new scientific advancements, which is discussed in detail in the Selectivity section below, and provide a standalone definition for prebiotic effect. Before submission, this paper was shared with key opinion leaders and external stakeholders for feedback and based on their comments, it was edited and agreed upon by GPA’s Board of Directors, Scientific & Technical and Regulatory Committees, and all associated Working Groups.

## The History of Prebiotics

### History of the prebiotic definition

The use of food components to modulate the gut microbiome and confer health benefits dates to the early 20th century [47], and in 1921, the specific microbiota-enrichment effects of carbohydrates on lactobacilli strains were described [48]. In 1995, Gibson and Roberfroid introduced the term prebiotic, and despite being initially considered as broadly defined, subsequent publications suggested that only a few nondigestible food components, including inulin, fructooligosaccharides (FOS), and galactooligosaccharides (GOS) fulfilled the definition criteria, which could be attributed to the viable quantification methods at the time.

Until the early 2000s, studying the GI microbiome was challenging, as several commensal bacteria are obligate anaerobes and difficult to culture under laboratory conditions. However, recent technological advancements such as next-generation sequencing and shotgun metagenomics made it possible to establish a data set library of human microbial communities and have an individual’s gut microbiota easily sequenced [4]. In the following years and by leveraging the newest available evidence, multiple groups attempted to update the 1995 definition [37,40,41].

In 2015, Bindels et al. [35] brought forward the following definition: “A prebiotic is a nondigestible compound that, through its metabolization by microorganisms in the gut, modulates composition and/or activity of the gut microbiota, thus conferring a beneficial physiological effect on the host.” By taking the concept of selectivity away from the prebiotic definition, they were moving it toward “ecological and functional features of the microbiota more likely to be relevant for host physiology,” such as the production of short-chain fatty acids (SCFAs), which act as signaling molecules to influence diverse systems of the body (e.g. immune, endocrine, and respiratory) [49,50].

In 2017, and more recently in 2024, ISAPP published an updated prebiotic definition [42] and scientific perspective [51], which resulted from the work of a panel of experts in nutrition, microbiology, and clinical research. This definition also applied to animals as it considered microbiota-focused strategies to maintain health and prevent diseases relevant to animals as they are for humans. While ISAPP’s definition was inclusive of novel prebiotic types like polyphenols and distant microbial sites such as the skin, it was limited as it retained the term selectivity, which was defined as the selective utilization of a prebiotic by a few but not all resident taxa [42]. Therefore, GPA aims to address the perspectives that have emerged in the years following ISAPP’s 2017 definition [51], not only updating the term prebiotic itself but also accounting for prebiotic effect.

### Regulatory definitions of prebiotics

To date, there is an absence of a globally unified definition for prebiotics. Most jurisdictions have different regulatory pathways for prebiotic ingredients and associated labeling. These requirements can vary significantly from country-to-country, making the process and category challenging to navigate for the industry, as well as health care professionals and consumers searching for prebiotic products in the marketplace.

In 2008, FAO held a Technical Meeting to revisit prebiotics, specifically addressing advancements within the field and discussing applications for human health, and ultimately defining a prebiotic as “a nonviable food component that confers a health benefit on the host associated with modulation of the microbiota” [39]. FAO’s definition addresses 3 key pillars, including component (i.e. prebiotics as a chemical substance), health benefit (i.e. prebiotics must have a measurable health effect), and modulation (i.e. prebiotic effect via modulation of the microbiota). This definition dropped selectivity and eliminated GI terminology when referring to the microbiota, permitting novel prebiotic formulations that elicit effects on distant microbial sites outside of the gut to be within scope [39].

In terms of established regulations, the United States FDA does not have an established prebiotic definition [44,52]. Instead, falling back on the 1995 definition by Gibson and Roberfroid, the United States National Center for Complementary and Integrative Health lists prebiotics under the “biologically based practices” domain [44]. The United States FDA regulates prebiotic use in foods just like other food ingredients, with 2 pathways available—as food additives or Generally Recognized as Safe. Food additives are substances that become components of food or affect food characteristics and require FDA premarket authorization. However, under the Generally Recognized as Safe framework, all safety data must be publicly available, the ingredient’s safe use must be widely acknowledged by qualified experts, and while premarket review is not required, it is recommended [53,54]. For dietary supplements, notifications of structure-function claims substantiated by human clinical trials are voluntary and do not require premarket approval. Nonetheless, such claims must be notified to the FDA if they are made on prebiotic dietary supplements, as they require competent and reliable scientific substantiation. However, if prebiotics are used to cure, alleviate, treat, diagnose, or prevent disease in the United States, they are classified as drugs and must go through the respective development and approval process.

In Canada, prebiotics that are part of Natural Health Product (NHP) formulations require scientific substantiation as per Health Canada’s Standards of Evidence. Additionally, labeling conventional foods with the term prebiotics is considered an implied health claim. While Health Canada acknowledges that prebiotics can include fiber, and dietary fiber claims do not require Health Canada review, other health claims that are disease-related or therapeutic in nature are subject to mandatory premarket review by Health Canada’s Food Directorate [55]. NHP and Veterinary Health Product prebiotic health claims are acceptable, depending on the source and substantiating evidence, with a list of available precleared health claims available for NHPs [56,57]. For example, inulin is currently the only ingredient in the category with precleared prebiotic claims in the form of a monograph [58]. Inulin is a commonly used and widely researched prebiotic, with sufficient scientific evidence supporting its safety, efficacy, and recommended conditions of use according to Health Canada’s Standards of Evidence and monograph threshold. The availability of precleared claims for inulin is helpful for inulin suppliers and formulators but limiting for other recognized prebiotic ingredients in terms of leveraging this fast-tracked product license application pathway to market. A product license application is a formal dossier that applicants must prepare, submit, and receive approval from Health Canada before selling and marketing finished NHPs. Since the time of Inulin Monograph publication, more prebiotics have become established through clinical research, which prebiotic-containing NHPs have leveraged to obtain Health Canada Product Licences outside of the monograph pathway. Thus, it is likely that other ingredients will be granted a similar status to that of inulin in the near future.

The Food Standards Australia and New Zealand is the regulatory body that oversees food claim requirements in Australia and New Zealand and allows for self-substantiated dietary fiber claims. Under Standard 1.2.7, Nutrition, Health and Related Claims, companies can make general-level health claims using publicly available evidence on the ingredient, without seeking preapproval from the Food Standards Australia and New Zealand [59]. In New Zealand, members of the Ministry for Primary Industries examine dossiers submitted, and those that fail to meet the ministry requirements are advised that the claim is not substantiated and may not be used [60]. Similarly, Brazil requires mandatory premarket review and clinical data for claims substantiation and approval. With this framework in place, the Brazilian regulatory agency, Agência Nacional de Vigilância Sanitária, has approved some ingredients as sources of dietary fibers to support gut function; among them are inulin from *Cichorium intybus* and FOS [61].

Lastly, the European Food and Safety Authority (EFSA), the regulatory authority in the EU, has no established prebiotic definition. EFSA does, however, recognize FAO’s prebiotic definition and requires supporting clinical trial data for health claim approval [62]. For example, EFSA has confirmed that the intake of 12 g/d of native chicory inulin has a scientifically demonstrated positive physiologic effect on bowel function [63]. Nonetheless, there is currently no harmonized position on the use of prebiotics in the region, and the approach often varies from one EU member state to another. With respect to country-specific regulations, Italy is among the leading countries to set national prebiotic guidelines. In 2013, the Italian Ministry of Health published a document on the use of prebiotics in foods and food supplements, referencing FAO’s definition [64]. Like Italy, many countries, including Malaysia, India, Colombia, Russia, and Argentina, have incorporated one of the previous definitions as part of their regional prebiotic regulations.

As a result of these global inconsistencies, a need arose within the industry to set international prebiotic guidelines and unified standards to discourage and eventually eliminate noncompliant practices. While GPA recognizes the complexity of setting regulatory guidelines and by echoing the requests in Sudan’s 2018 proposal to the Codex Committee on Nutrition and Foods for Special Dietary Uses, GPA anticipates working alongside international efforts to arrive at these regulations. GPA encourages regulators to continue evaluating the safety and efficacy of prebiotic ingredients and maintaining appropriate requirements for prebiotic classification and substantiation.

## GPA’s Prebiotic Definition

GPA’s updated definition describes a prebiotic as “a compound or ingredient that is utilized by the microbiota producing a health or performance benefit.” With respect to prebiotic classification, mechanism, location of action, and health and performance benefits, each concept will be discussed separately in the following sections.

### Established, novel, and emerging prebiotics

#### Overview

The original prebiotic definition from 1995 was limited to only a few carbohydrate-based food ingredients and focused on 2 bacteria (i.e. bifidobacteria and lactobacilli) as the target organisms [36,62,65]. Prebiotics that were originally discovered or shortly thereafter with a demonstrated effect on either or both bifidobacteria and lactobacilli were classified as traditional, or established, as they are referred to by GPA and in this article. Multiple types are currently recognized under this classification, including inulin, FOS, GOS, resistant starch (RS), and acacia gum. An increasing number of studies are also exploring novel (prebiotic candidates with increased research of their prebiotic efficacy) and emerging (under early investigation with potential for future use) prebiotic ingredients and have outlined an ever-expanding list of generally accepted, modifiable beneficial organisms within the microbiota beyond bifidobacteria and lactobacilli, which include *Akkermansia muciniphila*, *Ruminococcus bacilli*, *Faecalibacterium prausnitzii*, *Christensenellaceae* sp., *Bacillus subtilis*, and many more. Some of these novel and emerging prebiotic candidates include yeast-based substrates, botanicals, and amino acids [36,[66], [67], [68], [69]]. Prebiotics, whether established, novel, or emerging, work together to improve the microbiome in the following 3 general ways:

1) Support and feed: prebiotics act as substrates and food for microbiota, helping to support microbial activity and modulate composition.
2) Increase and influence: microbial metabolism of prebiotics contributes to the production of several metabolites, including SCFAs, which serve as energy reservoirs to promote the growth of additional microbes and increase microbial diversity.
3) Balance and optimize: prebiotics contribute to the optimization of the microbiome environment and help balance the levels of beneficial/harmful bacteria.

These mark the foundation of prebiotic mechanistic actions as seen in *in vitro*, animal, and human studies. Further to this, data from various prebiotic types point to different biochemical pathways being used to exert health benefits in the host. These benefits and their underlying mechanisms of action are discussed in the Health Benefits section.

#### Strategy for classification and substantiation

Several groups have proposed prebiotic classification criteria [21,37,39] that GPA considers appropriate for use, with terminology updates as necessary to maintain the clarity and applicability of the category and based on the current state of scientific knowledge. These criteria include the following: *1*) resistance to host digestive processes; *2*) utilization by the microbiota; and *3*) health benefit conferred via its microbiome-modulation effect. In addition to fulfilling these classification criteria, a prebiotic candidate must have its prebiotic effect substantiated by research. The amount of evidence then determines whether a prebiotic is established (i.e. extensively studied for its prebiotic effect), novel (i.e. increasing evidence of its prebiotic effect), or emerging (i.e. research is underway for its potential as a prebiotic). For example, acacia gum and RS are the 2 established prebiotic candidates that satisfy GPA’s prebiotic definition but have yet to be formally recognized as prebiotics by ISAPP. To combine efforts in setting clear criteria to characterize and classify prebiotic ingredients [51], GPA and its membership consisting of scientists and industry representatives are working toward creating Standards of Evidence to classify prebiotics, including existing and upcoming candidates.

The future of prebiotics will likely involve novel and emerging sources, such as isolated plant-based or synthetic prebiotics, which target specific microbial niches while focusing on trending issues such as sustainability, cost, and scalability. Sustainable sources of natural bioactive ingredients can be obtained from the billions of tons of food by-products and waste generated annually. Numerous side streams from fruits, vegetables, and grains contain potential prebiotics, such as pectic substances from orange peel and arabinoxylans from brewing waste, which can be extracted, purified, and marketed as prebiotic ingredients [36].

Expanding the classification of prebiotics beyond food and supplement ingredients is necessary given recent findings, which point toward medical and topical applications. While this article gives a few examples of prebiotic candidates (Table 1 and Supplemental Table 1) [26–29,33,43,66,70–98], it does not intend to classify all ingredients or products that can or will ultimately be labeled as a prebiotic. Such an initiative would first require the identification of prebiotic candidacy, then a comprehensive review of scientific evidence in the form of relevant and valid published clinical studies to substantiate the use of prebiotic candidates and their classification—much of which is already underway in parallel and equally extensive processes. Moreover, establishing an amalgamated and clear definition for prebiotics is a crucial predeterminant for the classification and integration of prebiotics within regulatory frameworks worldwide.

**TABLE 1:** Health benefit examples of established, novel, and emerging prebiotics in humans.

Prebiotic type	Established/novel/emerging^1^	Health effect/benefit	Dosage (per day) and formulation	Outcome	Reference	Jadad score and limitations
Acacia gum	Established	Glycemia	20 g powder mixed with hot water, tea, milk, or with food	Lowers fasting blood glucose response and increases satiety	[70]	**3.5**; none reported
		Dyslipidemia	30 mg tablets	Reduces cholesterol by 26%, LDL by 31%, and triglycerides by 38%	[71]	**1**; none reported
		Diabetes	30 g powder	Improves fasting and postprandial plasma glucose, postprandial insulin, glycosylated hemoglobin (HbA1c), total cholesterol, and triglycerides	[72]	**5**; study design flaw
			10 g powder mixed into lukewarm water		[73]	**1**; none reported
		Irritable bowel syndrome	10 g sachets mixed in orange juice	Significant improvement in stool frequency	[74]	**5** • Statistical power slightly underpowered • Study design flaw short study duration • Data analysis flaw no subgroup analysis or stratification
Amino acids	Emerging	Obesity	Two 15-g packs dissolved in water	Reduces levels of Bacillota (previously Firmicutes) and Actinomycetota (previously Actinobacteria)	[66]	**3**; none reported
Fructo-oligosaccharides	Established	Crohn disease	15 g sachets dissolved in water	Increases fecal bifidobacterial concentrations and mucosal dendritic cell function	[28]	**0**; none reported
		Constipation	6, 9, or 12 g in infant formula	Reduces bowel transit time and increases counts of *Bifidobacterium* spp.	[75]	**5** • Sample size insufficient • Research design limitation unexpected effect of secondary intervention
Galactooligosaccharides	Established	Behavioral changes	5.5 g powder mixed with water or food	Increases *Bifidobacterium* levels	[76]	**2** • Study design flaw appetite was not measured
		Irritable bowel syndrome	3.5 g & 7 g powder in sachets	Decreases flatulence, bloating and abdominal pain, improves stool consistency	[29]	**4.5**; none reported
		Immunity	5.5 g powder in sachets	Improves cytokine responses, natural killer cell activity and phagocytosis	[77,78]	**4**; none reported **4**; none reported
		Hyperpnea-induced bronchoconstriction	5.5 g Bimuno galactooligosaccharide supplement	Reduced airway inflammation markers	[79]	**5**; none reported
		Lactose intolerance	1.5–15 g in water	Improves lactose digestion and reduces abdominal pain	[80]	**5**; none reported
Human milk oligosaccharides (HMOs)	Established	Irritable bowel disease	5 or 10 g supplement	Gut microbiome modulation and mucosal response	[81]	**5** • Subject data limitation
Inulin	Established	Bone mineralization	8 g mixed with calcium-fortified orange juice	Enhances calcium absorption and whole-body bone mineral content and density	[82]	**5**; none reported
		Constipation	4 g mixed in a dairy product	Improves stool consistency	[83]	**5**; none reported
ω-3 Fatty acids	Novel	Cardiovascular markers	500 mg (165 mg EPA, 110 mg DHA, in gelatin capsules)	Gut microbiome modulation and short-chain fatty acid (SCFA) production	[43]	**2** • No placebo arm • Limited population mostly female subjects • Data collection techniques
		Stress	56 g of walnuts	Improves gut microbiome diversity	[33]	**3** • Potential sex bias in data analysis • No blinding • Differences in data collection conditions
Polyphenols	Novel	Diabetes	333 mg strawberry cranberry polyphenols (SCP) in a beverage	Improves insulin sensitivity	[84]	**4**; none reported
		Arterial stiffness	105.9 mg polyphenols from aronia berry capsules	Increases gut microbial richness and SCFA production	[85]	**4** • Population generalizability • Impact limitation no cause-effect relationship can be concluded
		Hypertension	500 mg polyphenolic blend (175 mg *Hibiscus sabdariffa* and 325 mg *Lippia citriodora*)	Reduce daytime parameters of blood pressure	[86]	**4** • Instrument limitations
Resistant dextrin (RD)	Novel	Type 2 diabetes	10 g RD (NUTRIOSE 06FM) powder supplement	Decreases fasting insulin, homeostasis model assessment of insulin resistance, quantitative insulin sensitivity check index, IL-6, TNF-α, endotoxin, and malondialdehyde	[87]	**5** • Sample size • Short intervention duration • Study design flaw gut or fecal microbiota were not assessed, nor were serum SCFA and nonesterified fatty acid levels
Resistant starch (RS)	Established	Glycemia	RS from high amylose corn (Hi-maize 260) and raw potato starch	Increases insulin sensitivity	[[88], [89], [90]]	**5** • Study size small • Study design flaw very restrictive exclusion criteria **4** • Sample size small • Study design flaw demanding study regimen **4** • Study technique/design flaw dietary fiber intake was not assessed and multiple relevant biomarkers were not measured.
		Type 2 diabetes	4.5 g RS from green banana	Decreases fasting glucose and HbA1c	[91]	**3** • Study technique/design flaw
		Cancer	30 g RS (1:1 blend of Novelose 240 and Novelose 330)	Substantial protective effects against noncolorectal cancers in patients with lynch syndrome	[92]	**4**; none reported
		Hypertension	40 g acetylated and butyrylated high amylose maize starch (HAMSAB) supplement	Increases SCFA production and lowers blood pressure	[93]	**5** • Sample size • Lack of long-term follow-up • Differences in the intervention vs. control diets
		Metabolic syndrome	20 g of green banana flour dissolved in milk, yogurt, or juice	Reduces blood pressure and fasting glucose	[94]	**1**; none reported
		Constipation	20 g Hi-maize RS2 high amylose corn starch delivered in biscuits	Constipation improved	[95]	**4** • Sample size small • The study population had a large age difference • Study design flaw short study duration
Xylans	Established	Obesity	Females: 25 g fermentable corn bran arabinoxylan Males: 35 g fermentable corn bran arabinoxylan	Increases propionate and *Bifidobacterium longum*. Enhances satiety and decreases HOMA-IR	[26,96,97]	**4.5** • Sample size • Study technique/design flaw
		Overweight and obesity	7.5 or 15 g arabinoxylan	Influences gene transcription, microbial diversity, fecal pH, and fecal SCFA concentrations	[98]	**5** • Study design flaw fecal SCFAs assessed and not intestinal SCFAs • Hepatic parameters not tested • Diet and exercise were not controlled
		Metabolic syndrome	15 g arabinoxylan supplement	Improves postprandial metabolic responses including serum glucose, insulin, and triglycerides	[27]	**2.5** • Sample size

1 Established: introduced with the discovery of prebiotics category or shortly after and have been extensively studied for their prebiotic effect. Novel: discovered recently and is increasingly researched as a prebiotic. Emerging: discovered recently and research is underway for its potential as a prebiotic.

### Selectivity

The concept of selectivity has been one of the most debated topics regarding the prebiotic definition over the years. Previously, several revisions tried to eliminate it [35,39], while others maintained it [21,41,42,99]. Although selectivity has been previously labeled as central to the prebiotic concept, the term is unclear within the context of the current prebiotic science. Hutkins et al. [62] highlighted the discrepancy in the dictionary definition of selectivity, which is “the property of affecting some things and not others,” and its applicability to prebiotic candidates. In 2017, ISAPP defined selectivity as the selective utilization of a prebiotic candidate by a few resident taxa and not all [42]. However, confusion persisted since some research groups used the term to mean effect instead of use. Furthermore, there has been no criteria stated for the concept of selectivity; in other words, how many taxa the prebiotic candidate must be used by and/or promote and to what extent (i.e. what is the minimum number or maximum number of species promoted) to be eligible for prebiotic classification [35,62]. In speaking with its membership comprised academics, dietitians, educators, formulators, marketers, and so on, confusion surrounding selectivity may be a hindering factor for product research and development and public awareness [62]. For this reason, GPA proposes to eliminate the term selectivity from the prebiotic definition to avoid confusion and arrive at a consensus definition between scientists, regulators, and industry, which can help drive the category forward and into the future.

Several prebiotic candidates fall within the nutritional category of dietary fiber and are regulated as such. Therefore, implementing unequivocal prebiotic regulations calls for a clear distinction between the 2 concepts—prebiotics and dietary fiber. Prebiotics and dietary fiber share common characteristics like digestion resistance and fermentability (at least for some fibers), but not all fibers can be classified as prebiotics or vice versa. As such, the use of the selectivity term has been attributed as a distinguishing factor for prebiotics from fibers [100]. However, most studied fibers, including nonstarch polysaccharides (e.g. pectin, inulin, and arabinoxylan), RS, and oligosaccharides (FOS and GOS) have been shown to have selective effects on the microbiota [[101], [102], [103]]. Furthermore, Wilson and Whelan [104] reported on commensal bacterial specificities for prebiotics. They referred to specific gene clusters within the bacterial genome dictating the bacterial enzymes responsible for metabolizing prebiotic substrates [104]. However, this group only used inulin-type fructans and GOS in their review, the same dietary oligosaccharides that Roberfroid [41] listed as the only prebiotic candidates that fulfill the classification criteria according to the concept of selectivity. In other words, inulin and GOS are the only prebiotic ingredients that satisfy the selectivity definition and are commonly classified as traditional prebiotics as both prebiotics are selectively fermented by beneficial bacteria, particularly species from the genera *Bifidobacterium* and *Lactobacillus*; however, they do not significantly promote the growth of pathogenic microorganisms, such as *C. difficile* or *E. coli* [35,41]. Modern research challenges the concept of prebiotic selectivity by showing that prebiotics affect a wider range of bacteria, interact through complex microbial networks, and produce effects influenced by individual variability and dose [24,62,105]. Novel nondigestible carbohydrates showing prebiotic potential are not as selective within the microbiome, potentially due to the functional redundancy among the gut microbiota and other mechanisms such as cross-feeding [35]. In 2013, Scott et al. [106] reported on cross-feeding, a process that enhances the prebiotic effect, defined as the product of one species being utilized by or having antagonistic effects on other species to confer a broader impact on the microbiome [24]. For example, *Ruminococcus bromii* is a keystone starch degrader that promotes the growth of *Anaerostipes hadrus*, a nonstarch utilizer, to produce butyrate [107]. Furthermore, Riva et al. [108] demonstrated how inulin may not be as selective as previously thought, identifying inulin-responsive bacteria. It was observed that diverse taxa from the phyla Bacillota (previously Firmicutes) and Actinomycetota (previously Actinobacteria) respond to inulin while *Coriobacteriia* sp., *Eggerthella lenta*, and *Gordonibacter urolithinfaciens* were indirectly stimulated by inulin’s degradation [108]. Therefore, the main distinguishing characteristic of dietary fibers compared with prebiotics is that not all dietary fibers are fermentable or interact with the microbiota to confer a health benefit, while prebiotics must possess these 2 characteristics to be classified as such [109,110]. Accumulating evidence over recent years has demonstrated prebiotic-induced modulation of various microbes, including *Clostridium coccoides*, *Clostridium leptum* groups, and *Bacteroides* sp., beyond the generally recognized probiotic strains bifidobacteria and lactobacilli [39,49]. Although ISAPP’s consensus paper from 2017 brought up the subject, noting that the insistence on selectivity does not mean excluding the effects on species other than lactobacilli and bifidobacteria [42,111,112], GPA recognizes that retaining the term in the current state of prebiotic science, industry, and regulations may be a factor of ambiguity and confusion, negatively affecting the category.

Both the FAO [39] and Bindels et al. [35] definitions dropped the concept of selectivity, mainly for being synonymous with the preferentially increased abundance of bifidobacteria and lactobacilli. Another reason for abandoning the term was the lack of general understanding on how selective the prebiotic effect needs to be to withhold the concept (i.e. how many taxa must the prebiotic enrich), unclear differentiation between beneficial and detrimental members of the gut microbiota, and increasing evidence revealing that a diverse microbiome is essential for intestinal homeostasis and health in general [35,39]. Thus, in the interest of all stakeholders, GPA is retiring the term selectivity from its prebiotic definition as it limits market growth potential and is outdated in light of modern research findings and technological advancements.

Since it has been a challenge to reach a consensus on a definition for the category, GPA believes that instead of relying on unclear terminology (i.e. selectivity) that may hinder these efforts, it is dropping the term selectivity and using previously mentioned criteria in the Strategy for Classification and Substantiation section to characterize and classify a prebiotic.

### Health benefits of prebiotics

#### Confirmation of health benefits

A wide range of benefits have been demonstrated with prebiotics, depending on the specific ingredient and metabolic conditions of the host. Established, novel, and emerging prebiotics have been shown to confer various health benefits in both humans and animals, with a few summarized in Table 1 and Supplemental Table 1. Clinical trials are essential to confirm prebiotic safety and efficacy in humans. While animal research is needed to substantiate prebiotic benefits in respective species, results do not directly translate to humans. As such, animal studies should only be used to substantiate the use of prebiotic ingredients in the animal species or serve to provide mechanistic insights for human trials. Nonetheless, high-quality animal research can be indicative as preclinical evidence of a prebiotic’s potential benefits in humans, noting that this distinction is intended to draw a line for quality purposes and to solidify the guideline that health benefits must be proven in the intended species. Therefore, high-quality randomized clinical trials, treated as the gold standard in research, with valid methodologies, sample sizes, populations, and end points demonstrating significant results must be used for substantiating prebiotic use in humans. In this article, several randomized controlled trials studying the health benefits of different prebiotics have been summarized in Table 1, which vary in their quality as evaluated using the Jadad scoring system, a commonly used procedure that assesses the methodologic quality of clinical trials based on objective criteria, including randomization, blinding, and reporting of withdrawals [113].

#### Examples of health benefits: established prebiotics

Numerous prebiotics have been long recognized for their prebiotic effect and, as such, are commonly known as established, which include acacia gum, inulin, FOS, GOS, and RS. Each of these have numerous demonstrated health benefits within the host, whether it be human or animal. For example, 10 g/d of acacia gum has been shown to enrich *Bifidobacterium, Lactobacillus*, and *Bacteroides* spp. [114], and cause a clinically meaningful increase as per FDA guidelines on stool frequency in constipation-predominant irritable bowel syndrome [74]. Higher doses of 20 and 40 g of acacia gum have also been effective at lowering blood glucose [115]. As such, proper communication around efficacious dose is critical to meeting the definition of a prebiotic and adhering to jurisdictional prebiotic regulations.

Oligosaccharides were the first ingredient category to be classified as prebiotics [35,41]. These prebiotics have been shown to increase bone mineral density in adolescents following consumption of 8 g/d of short-chain and long-chain inulin-type fructans [82], which have been attributed to their microbiome modulation effects of decreasing pH and improving GI permeability [116]. While individual prebiotics are usually of interest, mixtures of prebiotics have also demonstrated combined health benefits. For example, a mixture of 8 g/d of GOS and FOS consumed for 6 mo reduced the incidence of allergies 5 years later in high-risk infants [117]. In animals, this was also observed in the offspring of pregnant and lactating mice consuming a nondigestible GOS–inulin mixture by modulating the microbiota of mothers and their offspring [118]. In humans, FOS and GOS mixtures have been demonstrated to help control neonatal hyperbilirubinaemia in healthy, term infants [119], reduce the incidence of atopic dermatitis [120], and show preventative effects on infantile colic [121]. GOS as an individual ingredient has also been shown to improve GI symptoms such as abdominal pain, bloating, and flatulence in healthy individuals and those experiencing irritable bowel syndrome [29,32].

Other prebiotics such as RS have been shown to increase insulin sensitivity and reduce the risk of type 2 diabetes [122]. In 2016, the United States FDA considered qualified health claims for high-amylose maize RS as a dietary fiber following a petition submitted by ingredient manufacturer Ingredion Incorporated, which presented credible scientific evidence to substantiate the relationship between this food ingredient and a reduced risk of type 2 diabetes [123,124]. Studies have shown improved insulin sensitivity, which may be linked to microbiome modulation, in individuals with and without insulin resistance using 15–30 g/d of RS [123,88,89,100,125]. In animals, RS fermentation in the large intestine changed the expression of >200 genes in a rat model, including those believed to be involved in the regulation of peripheral insulin sensitivity [126]. In humans, RS was shown to reduce upper GI cancer in individuals with Lynch syndrome [92], reduce liver triglycerides in individuals with nonalcoholic fatty liver disease [127], reduce blood pressure in hypertensive patients [93], and reduce biomarkers of inflammation and oxidative stress [128]. The underlying mechanisms behind these prebiotics’ health benefits are discussed in more detail in the Mechanisms and Applications section.

Established prebiotics are more extensively studied than novel and emerging prebiotics in both humans and animals and have a wealth of scientific reports available for their numerous health benefits.

#### Examples of health benefits: novel prebiotics

Novel prebiotics such as resistant dextrin (RD), polyphenols, and ω-3 fatty acids have also been studied for their health effects. RD is recognized as a novel prebiotic for increasing satiety and improving both insulin resistance and determinants of metabolic syndrome [129]. RD has been the focus of many *ex vivo*, preclinical, and human studies. Notably, a randomized controlled clinical trial found that supplementation with 10 g/d of RD in women with type 2 diabetes led to significant decreases in fasting insulin, HOMA-IR, IL-6, TNF-α, malondialdehyde, and endotoxin concentrations compared with the placebo group [87]. More recently, an *ex vivo* study evaluated the indirect effects of RD supplementation on host immune response and gut barrier integrity and found that RD significantly increased transepithelial electrical resistance, increased levels of anti-inflammatory cytokines (IL-6 and IL-10), decreased levels of a proinflammatory cytokine (IL-8), increased the production of SCFAs, and enriched SCFA-producing bacteria [130]. These study results are indicative of RD’s many health benefits and enhanced gut barrier integrity and health effects.

Equally important, recent studies indicate the potential benefit of microbiome modulation on neurologic disorders through the gut–brain axis; as such, prebiotics may play a more significant role in cognitive health and function than previously thought, with further studies needed to confirm their exact benefits and mechanisms [[131], [132], [133], [134]]. In an animal study using a canine model, food supplemented with both polyphenols and ω-3 fatty acids demonstrated a positive effect on metabolites previously linked to anxiety [135]. However, additional research is needed to determine whether supplementation reduces anxiety-related behaviors in dogs, as well as in humans. A mouse study using desaminotyrosine, a specific phenolic metabolite, showed protective effects against influenza pathogenesis by enhancing type I interferon signaling. Interestingly, desaminotyrosine is only formed through the metabolization of flavonoids by a specific human-associated gut microbe, *Clostridium orbiscindens* [136]. Moreover, polyphenols interact with brush border enzymes, inhibiting starch breakdown, which slows down starch digestion and increases the proportion of RS reaching the large intestine [137]. Polyphenols can improve endothelial function in healthy men [138], stimulating the growth of probiotic organisms and increasing the production of SCFAs, including butyrate [23]. Human trials using a high-polyphenol Mediterranean diet have shown neuroprotective effects on age-related brain atrophy [34], along with other health benefits such as preventing weight regain, retaining glycemic control [139], and favorable changes in cardiometabolic biomarkers [140].

#### Examples of health benefits: emerging prebiotics

Emerging prebiotics are ingredients or substances recently shown in *in vitro*, animal, or human studies to possess prebiotic potential. Some commonly known emerging prebiotic candidates include amino acids, such as tryptophan, which has been shown in animal models to influence immune and nervous systems with links to the gut microbiome [141,142]. In addition, yeast hydrolysates, and fungal glycans are also gaining recognition as emerging prebiotics; however, human clinical trials are needed to confirm their candidacy.

Nonetheless, research is catching up for novel and emerging prebiotics, and new scientific evidence, including clinical and preclinical data, is slowly but steadily stacking up for their health benefits. The various types of recognized and emerging prebiotics exhibit benefits in inflammatory bowel disease, obesity, diabetes, allergies, colon cancer, neurologic conditions, and cardiovascular diseases through a range of mechanisms. Prebiotic mechanisms are either direct or downstream, supporting bacterial proliferation and metabolite production, consequently affecting the gut environment and host gene expression [1].

#### Mechanisms and applications

The classic mechanism of action is via prebiotic fermentation by various bacterial groups within the microbiome, enhancing the growth and metabolic activity of these and other commensal bacteria [36]. Additional mechanisms of action include immunomodulation, pathogen adhesion blockage, and mucus layer stimulation [[143], [144], [145], [146]].

Advances in the field of nutrigenomics made it possible to study the metabolites produced by fermentation and their effects on gene expression in the large intestine [126]. These metabolites include folate, indoles, secondary bile acids, trimethylamine-*N*-oxide, phenolics, and SCFAs [1]. As previously mentioned, the main by-products of the bacterial metabolism of prebiotics are SCFAs such as acetate, propionate, and butyrate, which are well-recognized, biologically active, and nutrigenomic agents [36,147]. Butyrate, specifically, provides the main energy source for colonocytes [148]. As acids, SCFAs are responsible for modifying the gut environment by decreasing its pH. SCFAs are then absorbed into the bloodstream through enterocytes, extending their effects to distant organs [24]. The concentrations of these metabolites along with changes in microbial composition impact numerous host systems including epithelial, immune, nervous, and endocrine signaling and impart various health effects such as improvements in bowel function, immune response, inflammation, glucose and lipid metabolism, bone health, and regulation of appetite and satiety [36]. Polyphenols produce phenolic compounds when fermented by the microbiome [149]. Catechins, anthocyanins, and proanthocyanidins have been shown in both human and animal studies to increase the relative abundance of probiotic bacteria such as *Lactobacillus*, *Bifidobacterium*, *Roseburia*, *Faecalibacterium*, and *Akkermansia* species and reduce pathobionts such as *E. coli*, *Clostridium perfringens*, and *Helicobacter pylori* [10,23,[150], [151], [152]]. Additionally, studies have investigated specific types of polyphenols. For example, flavanol, a polyphenol found in fruits, vegetables, and beverages like tea and red wine, as well as isoflavones, a plant estrogen found in soy, exert neuromodulatory and anticancer effects, respectively [10]. Resveratrol, another polyphenol from grapes and berries, has been evaluated to be used as a therapeutic substance for treating neuropathology and GI dysfunction [153]. Resveratrol has also shown potential when combined with probiotics such as *Bifidobacterium longum* for the treatment of obesity and nonalcoholic fatty liver disease [154].

In terms of prebiotic application and formulation, many types, delivery formats, doses, health benefits, and combinations are available [36]. Prebiotics are sometimes synthetically made to meet large-scale consumer and production demands for dietary supplements, functional foods, and medical foods [24]. With high pressure for speed-to-market, more companies are taking risks and releasing prebiotic products with false or inaccurate claims that are not scientifically valid. To protect consumers against products carrying misleading claims, GPA encourages the implementation of transparency processes. One such initiative is NutraStrong™ Prebiotic Verified, a certification program specific to prebiotics that addresses industry concerns by maintaining a level of quality standardization, including verification of input quantity and ingredient efficacy provided from either the supplier or internal sources [155].

While consumer awareness of prebiotics is increasing year over year, gaps remain around the variety of their health benefits, mechanisms of action, sources, and the definition of prebiotics itself [36]. With the current understanding of prebiotic health and performance benefits and actions on the body’s various microbiomes, it is only appropriate for the prebiotic definition to be updated to reflect recent knowledge.

### Performance benefits of prebiotics

Performance can be considered in broader contexts, referring to physical and mental capability in humans or animals. Increasing research shows promising results in using prebiotics as targeted human and animal therapies for performance improvement. In animals, prebiotics have demonstrated positive effects in horses, enhancing gut microbiota production of SCFAs, preventing lactate accumulation, lessening digestive stress, and improving performance [46,156,157]. Prebiotics alone or when paired with probiotics have also shown benefits in dogs, including sled dogs, such as improving fecal scores, reducing cases of diarrhea, decreasing proteolytic fermentation, and modulating the immune system [[158], [159], [160], [161]]. In addition, oligofructose-enriched inulin has shown promise in preclinical settings in male Wistar rats to improve cognitive performance and learning ability [162,163]. While the science behind the benefits of prebiotics on animal performance is promising, more research is needed.

In humans, most of the prebiotic performance research has focused on cognition. The American Psychological Association defines cognitive performance as the execution of mental processes such as perception, learning, memory, understanding, awareness, reasoning, judgment, intuition, and language [164]. The emergence of nutritional neuroscience has advanced the general understanding of the bidirectional communication channel between the brain and the gut microbiome, commonly known as the gut–brain axis [45,165]. Preliminary evidence from nonclinical and clinical trials supports the microbiome’s essential role in the development and progression of various mental disorders, including, but not limited to, anxiety and depression [[165], [166], [167]], and the use of dietary microbiome-modulating interventions such as prebiotics for improving cognitive performance and mental disorders [33,45,166]. Previous reports have correlated advancing age with declining microbiome diversity and cognitive ability. Microbiome-modulating dietary interventions such as prebiotics may benefit cognition and the aging brain. A recent randomized, double-blinded, placebo-controlled study by Aljumaah et al. [168] highlighted specific gut microbiome members correlated with cognitive performance, specifically mild cognitive impairment in 169 middle-aged and older adults who received 3-mo intervention (*Lactobacillus rhamnosus* GG probiotic or placebo). The study identified these members to be *Prevotella ruminicola*, *Bacteroides thetaiotaomicron*, and *Bacteroides xylanisolvens* and their lower relative abundance linked to an improved cognitive score [168]. Similarly, Hu et al. [167], using metagenome sequencing of stool samples of mild, moderate, and severe major depressive disorder patients, reported high *Bacteroides* sp. abundance, which positively correlated with the severity of major depressive disorder, and depleted *Ruminococcus* and *Eubacterium* spp. As such, these taxa may be targeted for manipulation by dietary, microbiome-modulating interventions such as prebiotics to improve cognitive performance, not only in aging individuals but also in other age groups.

Lastly, athletic performance is another area of interest with respect to prebiotic performance benefits in humans. Generally, athletes consume nutritious foods to optimize health and foster peak performance, in addition to prevent disease [169]. Research has highlighted prebiotic benefits in athletic performance via gut microbiome modulation. The microbiome’s involvement in athletic practice potentially manifests through harvesting energy from the food digestion, fighting off pathogens, enhancing hydration [170], and supporting the immune system [171,172]. Regarding the immune system, the anti-inflammatory and antioxidant properties of prebiotics may support the body’s intrinsic processes to regulate inflammation and maintain it as a healthy response to exercise, providing adequate postexercize recovery and performance [171]. Prebiotics may also play a role in reducing the risk of upper respiratory tract infections and GI tract issues [173,174]. With fewer respiratory infections, athletes would not lose training time to sickness, and performance may also be maintained [79,175]. Furthermore, prebiotic fermentation is beneficial by its contribution to the production of SCFAs [171], which can be utilized locally within the colon for energy generation or transferred via the bloodstream to skeletal muscles. In skeletal muscles, SCFAs have various mechanisms that influence performance, including increasing the bioavailability of glucose, glycogen, and fatty acids during exercise [169].

Prebiotics may be effectively utilized in the future for enhancing performance benefits via their microbiome-modulatory effects. High-quality research in both humans and animals is expected to continue, resulting in more published literature in the near future.

### Sites of action

Oral formulations have been the most used route of administration in prebiotic research to influence the gut microbiome. Nevertheless, different niches across the human body harbor microbial communities besides the gut, including the oral cavity, skin, urogenital tract, and respiratory system. Recent research reports many nonoral (e.g. topical) applications of novel prebiotic formulations that contribute to human health through prebiotic–microbiome interactions. Several microbiome sites are introduced in the following sections, where some novel prebiotic formulations are demonstrating effects [146,[176], [177], [178]].

#### Microbiome of the GI tract

The GI or gut microbiome is the most studied among microbiome locations in the body. As discussed earlier, the GI tract has a rich and diverse microbial community centered in the colon, with the reported number of species ranging between 400 and 1500 different species [3,8]. As a functional ecosystem within the body, this microbial community requires high levels of diversity to maintain working relationships among its members, contributing to health and well-being. However, disruptions to microbiome diversity, composition, and function have been associated with various metabolic, allergic, and other health conditions. While the makeup of the gut microbiome in human adults is relatively stable, certain components are metabolically flexible [179]. Besides the host’s genetics, the gut microbial composition is influenced by numerous endogenous (i.e. age, gender, hormonal changes, and health status) and exogenous (i.e. diet, drugs, exercise, and environment) factors [1,10]. The GI microbiome is interconnected with other microbiome sites and the organs and bodily systems it interacts with; therefore, prebiotic actions on the gut microbiome may influence these systems (e.g. via SCFA).

#### Microbiome of the oral cavity

The oral cavity microbiome contains hundreds of species that protect against extrinsic bacterial colonization and guard systemic health [180]. These species are estimated at a minimum of 700 from ≥12 phyla and include archaea, but most remain uncultured due to their need for very specific conditions including extreme oxygen sensitivity and dependence on neighboring organisms [180]. Dysbiosis in the oral microbial ecosystem has been linked not only with local oral disease but also with numerous systemic diseases, including cardiovascular, metabolic, respiratory, neurologic, and cancer [133,181]. The investigation of therapeutic applications for prebiotics and oral microbiome modulation is currently underway [133,134] with multiple prebiotics tested. For example, *N*-acetyl-D-mannosamine has shown promising results in *in vitro* studies to trigger the growth of beneficial oral bacteria [182].

#### Microbiome of the skin

The human skin is one of the largest and most versatile organs of the human body. The human skin microbiome, which comprised ∼10^8^–10^10^ colonizing microbes in a healthy adult [183], has adapted to the skin’s dehydrated, nutrient-poor, acidic environment, maintaining the skin barrier and immune response, and preventing pathogenic growth. Similarly to the gut microbiome, dysbiosis in the skin microbiome has been linked to skin conditions, whereas a diverse skin microbiome supports skin integrity and homeostasis [183]. For example, prebiotic glucomannan hydrolysates have been shown to impart skin microbiome-modulating effects and reduce acne when administered topically [184]. Colloidal oat-containing lotion directly affects the growth, metabolism, lactic acid production, and gene expression of skin commensal bacteria and has been proposed as a topical prebiotic [176].

#### Microbiome of the urogenital tract

The urogenital tract microbiome is a complex and dynamic system of >200 bacterial species, mainly dominated by lactobacilli [185]. The makeup of the urogenital tract microbiome is influenced by sex, genes, ethnic background, environmental (e.g. pH), and behavioral factors. While vaginal microbiomes of most women are dominated by *Lactobacillus* sp., around 25% of women in North America have shown communities that are composed of a more proportionally even community of obligate and facultative anaerobes. This condition is termed bacterial vaginosis and has been associated with a higher incidence of urogenital infections [186]. Novel therapies such as topical applications of maltose gel have been used to treat bacterial vaginosis by promoting a shift in the vaginal microbiome from vaginosis-related dominant bacteria to a *Lactobacillus*-dominant microbiome [178]. Further research is needed to confirm the prebiotic potential of maltose gel and other novel therapies along the urogenital tract.

#### Microbiome of the respiratory tract

The respiratory tract has an ideal environment with favorable temperature, moisture and mucus, and a large surface area that is in constant contact with the external environment, making this microbiome more transient and dynamic than the other microbiome locations [187]. As such, the respiratory tract microbiome plays an important role in the protection against the colonization of extrinsic harmful bacteria that could affect systemic health. The respiratory microbiome provides cues to the host immune system, which appear to be vital for the maintenance of immune tolerance against viral and microbial respiratory infections [[187], [188], [189]]. Although the direct use of prebiotics on the respiratory tract microbiome has not yet been explored, some oral prebiotics influencing the gut microbiome have conveyed therapeutic benefits to the respiratory tract [175,177,190]. For example, oral GOS showed immunomodulatory effects in asthma patients, highlighting its potential to modulate the underlying immunopathology of asthma [79]. Furthermore, FOS and GOS modulated the gene expression of cytokines and inflammatory markers in a mouse model [146]. Therefore, future innovations may further elucidate prebiotic effects in this microbiome niche.

## GPA’s Definition of Prebiotic Effect

### Defining the prebiotic effect

In addition to defining a prebiotic, GPA went a step further to distinguish prebiotic effect. This standalone portion is needed to reinforce the difference between prebiotics and fibers, defining prebiotic effect with respect to health and wellness promotion. Historically, prebiotic effect has referenced changes in gut microbiome composition and specific (patho)physiologic effects in experimental and human intervention studies [65]. Despite being previously defined by Bindels et al. [35] as “the beneficial physiological outcome that arises from the modulation of the composition and/or activity of the gut microbiota through the metabolization of a nondigestible compound,” there has been no formal recognition of the term.

GPA, aiming to maintain prebiotic product integrity, efficacy, and transparency within the category, acknowledges that a distinct definition for the prebiotic effect is fundamental. GPA defines prebiotic effect as “a health or performance benefit that arises from alteration of the composition and/or activity of the microbiota, as a direct or indirect result of the utilization of a specific and well-defined compound or ingredient by microorganisms.”

This definition will serve as a tool to further regulatory discussions and policymaking, potentially being used by agencies to set guidelines and language around claims and labeling. Specifically, the definition can distinguish between ingredients as prebiotics and those with prebiotic effects, which is important when it comes to labeling and only possible through clearly differentiating the 2 concepts. Therefore, a clear, formal, and inclusive definition of prebiotic effect could increase overall awareness, usefulness, and applicability within the category.

### Demonstrating a prebiotic effect

Prebiotic effect is typically associated with the production of SCFAs and other metabolites [75,191] or bacterial mechanisms like cross-feeding [106]. Prebiotic effect can vary between different prebiotics, being influenced by the food source, chemical structure of the compound, or individual differences in gut microbiome composition [23]. In 2010, Roberfroid et al. [65] stated that “A prebiotic effect exists and is now a well-established scientific fact. A large number of human intervention studies have demonstrated that dietary consumption of food products/ingredients/supplements results in statistically significant changes in the composition of the fecal (and in some cases, the mucosal) gut microbiota.”

Defining prebiotic effect separately from the prebiotic definition is crucial to product labeling as deeming some compounds as solely prebiotics can be limiting in terms of failing to describe their roles within the body, which may extend beyond gut microbiome modulation [35]. In previous sections, several established, novel, and emerging prebiotics were listed for their various health benefits. While these compounds have different roles within the body, their microbiome-modulation activity and subsequent health benefits may manifest via their prebiotic effect. Polyphenols, for instance, are a diverse group with multiple classes, including flavonoids, tannins, and phenolic acids [23]. Previously, polyphenols’ most well-known health benefits were associated with their antioxidant and anti-inflammatory properties. However, recent reports have demonstrated that different polyphenol classes impart health benefits as prebiotic substrates [10]. Thus, labeling polyphenols as only prebiotics, as opposed to highlighting their prebiotic effect, is limiting to their wide range of health benefits, especially when considering commercial and health applications.

Prebiotic effect should ideally be substantiated by scientific evidence that demonstrates the effect is causally linked to specific changes in the microbiome, conferring a measurable and observable health benefit. Although statistical and humanized gnotobiotic approaches can be used to provide evidence for causal associations, causality between prebiotic consumption and specific health outcomes is challenging to prove in human studies, which provide primarily correlative evidence [51]. Bustamante et al. [192] recommended that the prebiotic activity of an ingredient should be evaluated by a series of *in vitro* and *in vivo* assays with the following proposed methodologies: *1*) *in vitro* digestion and *in vitro* fermentation, *2*) *in vivo* human and animal studies, *3*) analysis of fermentation products, and *4*) validation of *in vitro* with *in vivo* studies. However, data from animal models have major limitations due to biological discrepancies between species. Scott et al. [193] reported that prebiotic effects do not always translate mechanistically from animal to human studies, or when they do, are limited to subgroups in whom dietary, microbiome, or individual characteristics create the ideal environment. Preclinical studies using appropriate animal and cell models provide preliminary insight into the mechanisms of prebiotics, which could then be tested in humans and substantiated by the resulting data. Additionally, as causation is challenging to demonstrate in relation to gut microbiome changes in humans, most human studies aim to produce correlations or associations instead [194]. At minimum, relevant randomized controlled trials with comprehensive inclusion/exclusion criteria, adequate sample sizes, and validated end points should be consolidated as proof of an association between the microbiome-modulatory effect and measurable physiologic and health benefits [35,39]. In fact, randomized controlled trials, using randomization to minimize biases, are considered the gold standard for evaluating an effect, and typically, multiple trials that replicate the observations are required to arrive at an extrapolated causation conclusion [195]. As such, researchers continue to ascertain how generalizable and durable prebiotic effects are across human populations of different ages and physiological states [22].

Therefore, prebiotics for use by humans must be characterized using substantiating evidence from human clinical trials linking physiologic effects to gut microbiome alterations, while animal studies could provide mechanistic and causal insights into prebiotic actions in humans in addition to their use for substantiating prebiotic use in animals. If this approach is implemented in a regulatory setting along with the previously mentioned criteria in characterizing prebiotic candidates, it may eliminate current trends of products carrying claims that are scientifically weak due to gaps in the availability of human data and challenges around translating results from animals to humans.

## Goal and Target Audience of the GPA

Given the widespread interest in prebiotics and their effects, having an expanded definition is important not only to the scientific community but also to consumers, health care professionals, industry, and governmental agencies [62]. GPA intends for this definition to be leveraged by stakeholders, using it for formulation, consumption, administration, claims substantiation, and/or for review and approval purposes, without confusion and with scientific confidence as it closely follows the previous definitions from ISAPP [42] and Bindels et al. [35].

While technical distinctions may be useful for researchers and those knowledgeable in the space, it may not translate to a lay audience. A survey of 245 health care professionals, including 100 physicians, showed that only 22% were familiar with the term prebiotic [196]. Similarly, another survey of American adults in an inpatient urban hospital setting revealed that 11% were aware of the term and only 7% were able to select the correct definition from 4 other choices [197]. There is clearly a need to raise awareness and develop an understanding that spans the spectrum of technical advancements. GPA champions initiatives to raise awareness and increase education about prebiotics, but with concerns and confusion surrounding the previous definitions, GPA is proposing a separate, more generic prebiotic definition and an advanced description of prebiotic effect as both remain accurate and necessary to fulfill distinct needs. Furthermore, familiarity is rising, as GPA-led survey-based consumer data show that even among health care professionals, a simpler, more easily communicated yet scientifically valid and allowable for emerging developments (e.g. performance benefits associated with prebiotics) definition is actively being sought. With the inclusion of performance benefits, scientists may be encouraged to increase their research in this area, consequently supporting product innovation in this space to meet consumer needs.

While reaching consensus on a new definition may not entirely resolve the debate surrounding prebiotics, a meaningful and well-considered change to the previous definitions that excludes the term selectivity and separates prebiotic and prebiotic effect is necessary to advance the category. A global consensus definition may also assist regulators when drafting prebiotic regulation and standards. As discussed, the industry is interested in a unified definition that enables companies to approach regulatory agencies with confidence in terms of substantiating product quality, safety, and efficacy. By building trust, consumption and innovation activities may increase, including the use of novel formats and formulations to meet consumer demand.

## Concluding Remarks

Despite the significant contributions and efforts from groups in the past, including FAO and ISAPP, products continue to be sold that carry the term prebiotic without meeting the respective jurisdictional requirements. While GPA does not intend to challenge prebiotic regulations, it recognizes their importance for driving the category forward by enabling the development of innovative and novel prebiotic products that satisfy consumer needs and reducing or eliminating the misconceptions associated with the category. As such, GPA and its expert communities recognize the need for an updated and unified definition that clearly characterizes and classifies prebiotic compared with prebiotic effect. This may lead to the creation of guidance documents and standardized procedures around clinical research, trial design, and regulatory oversight that contribute to prebiotic compliance globally. With the 2 new definitions, GPA aims to be comprehensive and clear, avoiding any forms of ambiguity, for the purpose of being easily leveraged by stakeholders, contributing to the ongoing debate, and enabling continued advancements within the category.

GPA defines a prebiotic as “a compound or ingredient that is utilized by the microbiota producing a health or performance benefit,” and prebiotic effect as “a health or performance benefit that arises from alteration of the composition and/or activity of the microbiota, as a direct or indirect result of the utilization of a specific and well-defined compound or ingredient by microorganisms.” Both proposed definitions are rooted in science to account for established, emerging, and novel prebiotics, are comprehensive in their explanation and justification for each part (i.e. what constitutes an ingredient or benefit), and are user-friendly for all potential stakeholders. GPA hopes that this definition will be used to enhance prebiotic research and consumer awareness and support product innovations that adhere to regional guidelines and substantiation requirements for prebiotic classification.

## Author contributions

The authors’ responsibilities were as follows—RSW, TJ, LM: were involved in the initial discussions from which the ideas for this paper were generated; ECD, SAA, RSW, PG: contributed written sections for the paper; SAA, PG: conducted the literature search and compiled all sections of the paper; and All Authors: have reviewed and approved the final manuscript.

## Conflict of interest

ECD was the recipient of the 2021 Young Researcher Award offered by GPA. ECD also serves as a Science & Technical Advisor to the GPA but has not received any monetary compensation for his contribution to the GPA or this manuscript. SAA, RSW, PG, and TJ are employed at companies that produce and sell or provide services involving probiotics and prebiotics (SAA, PG, and TJ: SGS Nutrasource; RSW: ADM).

## Funding

The authors reported no funding received for this study.
