# Supplementary material for: Revisiting the Concepts of Prebiotic and Prebiotic Effect in Light of Scientific and Regulatory Progress—A Consensus Paper From the Global Prebiotic Association
Source: Adv Nutr. 2024 Oct 29;15(12):100329. doi: 10.1016/j.advnut.2024.100329 (PMC11616045; doi:10.1016/j.advnut.2024.100329)
Supplement: Multimedia component 1 [file mmc1.pdf]

Supplementary Material

Revisiting the Concepts of Prebiotic and Prebiotic Effect in Light of Scientific and Regulatory Progress – A Consensus Paper from the Global Prebiotic Association (GPA)

Edward C. Deehan, Santa Al Antwan, Rhonda S. Witwer, Paula Guerra, Tania John, and Len Monheit

Table of Contents

- **Supplemental Table 1.** Health Benefit Examples of Established, Novel, and Emerging Prebiotics in Animals
- **Supplemental References.**

Supplemental Table 1: Health Benefit Examples of Established, Novel, and Emerging Prebiotics in Animals.

Prebiotic Type	Established/ Novel/ Emerging*	Health Effect/Benefit	Dosage (per day) and Formulation (Animal Model)	Outcome	Reference
Acacia Gum	Established	Renal toxicity	7.5 g/kg in oral gavage (Rat)	Reverses and prevents mercury-induced degenerative changes of the kidneys	(1)
		Renal function	20 g/kg body weight (Mouse)	Increases creatine clearance and alters electrolyte excretion	(2)
Amino Acids	Emerging	Immunity	Basal diet (containing 0.93% or 0.5% L-arginine g/g (Mouse)	Gut microbiome modulation and innate immunity activation	(3)
Botanicals (e.g., Fenugreek)	Emerging	Obesity	150 mg/kg body weight (Rat)	Anti-obesogenic properties	(4)
Fructooligosaccharides (FOSs)	Established	Metabolic disorders	0.3 g in tap water (Mouse)	Gut microbiome modulation	(5)
		Non-alcoholic steatohepatitis	5% FOS in drinking water (Mouse)	Gut microbiome modulation	(6)
Galactooligosaccharides (GOSs)	Established	Infection	1% w/w GOS in diet (Chicken)	Cecal microbiome modulation	(7)
Inulin	Established	Infection	0.5% or 1.0% inulin supplemented diet (Chicken)	Decreases the proinflammatory response and enhances mucosal immunity	(8)
		Hepatic steatosis	N/A (Rat)	Restores intestinal barrier integrity, rehabilitated lipogenesis regulators, and attenuated steatosis phenotypes	(9)
		Breast cancer	Diet pellets supplemented with 8% (wt:wt),10% (wt:wt), and 15% (wt:wt) inulin (Mouse)	Suppresses tumorigenesis, modulates microbial composition, and increases plasma propionic acid level that affect gene expression, cell proliferation, and cell survival	(10)

Omega-3 Fatty Acids	Novel	Stress	1.25% Fish oil or 3.12% microalgae supplemented soybean meal (Piglet)	Reduces inflammatory response	(11)
		Obesity	N/A (transgenic model with endogenous omega-3)	Improves antibiotic-induced gut dysbiosis	(12)
Polyphenols	Novel	Obesity	Grape powder (5% w/w) (Mouse)	Various effects on the metabolic consequences	(13)
			0.5% polymeric procyanidins (dose undeclared) (Mouse)	Regulation of lipid metabolism-associated genes and gut microbiota	(14)
		Influenza infection	N/A (Mouse)	Enhances type I interferon (IFN) signaling	(15)
		Chronic diseases	50 mg/kg berry extract in water solutions (Mouse)	Improves antioxidant status	(16)
Resistant Starch (RS)	Established	Eye health	Low glycemic diet with RS vs. High glycemic starch control (Mouse)	Reduces age-related macular degeneration	(17)
		Immune function	20% w/w high amylose maize RS (Mouse)	Modifies the intestinal microbiota, stimulates intestinal immunity and endocrine-responses, and modifies systemic metabolomes	(18)
		Chronic kidney disease	59% w/w high amylose maize-RS type 2 (HAMRS2) (Rat)	Gut microbiome and metabolomic profile modulation	(19)
Xylans	Established	Hypercholeste-rolemia	5 g/kg arabinoxylan in diet (Hamster)	Reduces cholesterol levels	(20)
		Immune function	6 g/kg isomalto-oligosaccharide supplemented diet (Pig)	Regulates gut microbiota and strengthens immune function	(21)
		Obesity	10% w/w arabinoxylan in high-fat diet (dose undeclared) (Mouse)	Decreases fecal markers of inflammation	(22)

Yeast Hydrolysates	Emerging	Growth efficiency	0.05% or 0.1% yeast hydrolysate supplemented diet (Pig)	Enhances growth performance, nutrient digestibility, and *Lactobacillus* count	(23)
		Performance	2g /kg yeast derivatives supplemented diet (Pig)	Gut microbiome modulation	(24)
Fungal Glycans	Emerging	Obesity	3 or 5 g/kg β-glucan in high-fat diet (HFD) (Mouse)	Regulates gut microbiota and ameliorates HFD-induced hepatic stress, colonic motility, and intestinal atrophy	(25)

*Established: Introduced with the discovery of prebiotics category or shortly after and have been extensively studied for prebiotic effect.
Novel: Discovered recently and is increasingly researched as a prebiotic.
Emerging: Discovered recently and research is underway for its potential as a prebiotic.

Supplemental References

1. Gado AM, Aldahmash BA. Antioxidant effect of Arabic gum against mercuric chloride-induced nephrotoxicity. *Drug Des Devel Ther*. 2013;7:1245-52.
2. Nasir O, Artunc F, Saeed A, Kambal MA, Kalbacher H, Sandulache D et al. Effects of gum arabic (Acacia senegal) on water and electrolyte balance in healthy mice. *Journal of renal nutrition : the official journal of the Council on Renal Nutrition of the National Kidney Foundation*. 2008;18(2):230–8.
3. Ren W, Chen S, Yin J, Duan J, Li T, Liu G et al. Dietary arginine supplementation of mice alters the microbial population and activates intestinal innate immunity. *J Nutr*. 2014 Jun;144(6):988-95.
4. Konopelniuk VV, Goloborodko, II, Ishchuk TV, Synelnyk TB, Ostapchenko LI, Spivak MY et al. Efficacy of Fenugreek-based bionanocomposite on renal dysfunction and endogenous intoxication in high-calorie diet-induced obesity rat model-comparative study. *EPMA J*. 2017 Dec;8(4):377-90.
5. Everard A, Lazarevic V, Gaia N, Johansson M, Stahlman M, Backhed F et al. Microbiome of prebiotic-treated mice reveals novel targets involved in host response during obesity. *ISME J*. 2014 Oct;8(10):2116-30.
6. Matsumoto K, Ichimura M, Tsuneyama K, Moritoki Y, Tsunashima H, Omagari K et al. Fructo-oligosaccharides and intestinal barrier function in a methionine-choline-deficient mouse model of nonalcoholic steatohepatitis. *PLoS One*. 2017;12(6):e0175406.
7. Hughes RA, Ali RA, Mendoza MA, Hassan HM, Koci MD. Impact of Dietary Galacto-Oligosaccharide (GOS) on Chicken's Gut Microbiota, Mucosal Gene Expression, and Salmonella Colonization. *Front Vet Sci*. 2017;4:192.
8. Song J, Li Q, Everaert N, Liu R, Zheng M, Zhao G et al. Effects of inulin supplementation on intestinal barrier function and immunity in specific pathogen-free chickens with Salmonella infection. *J Anim Sci*. 2020 Jan 1;98(1).
9. Yang Z, Su H, Lv Y, Tao H, Jiang Y, Ni Z et al. Inulin intervention attenuates hepatic steatosis in rats via modulating gut microbiota and maintaining intestinal barrier function. *Food research international (Ottawa, Ont)*. 2023;163:112309.
10. Wu H, Van Der Pol WJ, Dubois LG, Morrow CD, Tollefsbol TO. Dietary Supplementation of Inulin Contributes to the Prevention of Estrogen Receptor-Negative Mammary Cancer by Alteration of Gut Microbial Communities and Epigenetic Regulations. *International journal of molecular sciences*. 2023;24(10):9015.
11. Lee AV, You L, Oh SY, Li Z, Code A, Zhu C et al. Health Benefits of Supplementing Nursery Pig Diets with Microalgae or Fish Oil. *Animals (Basel)*. 2019 Mar 5;9(3).
12. Kaliannan K, Wang B, Li XY, Bhan AK, Kang JX. Omega-3 fatty acids prevent early-life antibiotic exposure-induced gut microbiota dysbiosis and later-life obesity. *Int J Obes (Lond)*. 2016 Jun;40(6):1039-42.
13. Collins B, Hoffman J, Martinez K, Grace M, Lila MA, Cockrell C et al. A polyphenol-rich fraction obtained from table grapes decreases adiposity, insulin resistance and markers of inflammation and impacts gut microbiota in high-fat-fed mice. *J Nutr Biochem*. 2016 May;31:150-65.
14. Masumoto S, Terao A, Yamamoto Y, Mukai T, Miura T, Shoji T. Non-absorbable apple procyanidins prevent obesity associated with gut microbial and metabolomic changes. *Sci Rep*. 2016 Aug 10;6:31208.
15. Steed AL, Christophi GP, Kaiko GE, Sun L, Goodwin VM, Jain U et al. The microbial metabolite desaminotyrosine protects from influenza through type I interferon. *Science (New York, NY)*. 2017;357(6350):498–502.
16. Chen J, Shu Y, Chen Y, Ge Z, Zhang C, Cao J et al. Evaluation of Antioxidant Capacity and Gut Microbiota Modulatory Effects of Different Kinds of Berries. *Antioxidants (Basel)*. 2022 May 22;11(5).

17. Rowan S, Jiang S, Korem T, Szymanski J, Chang ML, Szelog J et al. Involvement of a gut-retina axis in protection against dietary glycemia-induced age-related macular degeneration. *Proceedings of the National Academy of Sciences of the United States of America*. 2017;114(22):E4472–E81.
18. Barouei J, Bendiks Z, Martinic A, Mishchuk D, Heeney D, Hsieh YH et al. Microbiota, metabolome, and immune alterations in obese mice fed a high-fat diet containing type 2 resistant starch. *Molecular nutrition & food research*. 2017;61(11).
19. Kieffer DA, Piccolo BD, Vaziri ND, Liu S, Lau WL, Khazaeli M et al. Resistant starch alters gut microbiome and metabolomic profiles concurrent with amelioration of chronic kidney disease in rats. *Am J Physiol Renal Physiol*. 2016 May 1;310(9):F857-71.
20. Tong LT, Zhong K, Liu L, Qiu J, Guo L, Zhou X et al. Effects of dietary wheat bran arabinoxylans on cholesterol metabolism of hypercholesterolemic hamsters. *Carbohydr Polym*. 2014;112:1-5.
21. Wu Y, Pan L, Shang QH, Ma XK, Long SF, Xu YT et al. Effects of isomalto-oligosaccharides as potential prebiotics on performance, immune function and gut microbiota in weaned pigs. *Animal Feed Science and Technology*. 2017;230:126-35.
22. Neyrinck AM, Possemiers S, Druart C, Van de Wiele T, De Backer F, Cani PD et al. Prebiotic effects of wheat arabinoxylan related to the increase in bifidobacteria, Roseburia and Bacteroides/Prevotella in diet-induced obese mice. *PLoS One*. 2011;6(6):e20944.
23. Sampath V, Han K, Sureshkumar S, Kim IH. Impact of yeast hydrolysate (Saccharomyces cerevisiae) supplementation on the growth performance, nutrient digestibility, fecal microflora, noxious gas emission, blood profile, and meat quality of finishing pigs. *Canadian journal of animal science*. 2021;102(1):98-107.
24. Hasan S, Junnikkala S, Peltoniemi O, Paulin L, Lyyski A, Vuorenmaa J et al. Dietary supplementation with yeast hydrolysate in pregnancy influences colostrum yield and gut microbiota of sows and piglets after birth. *PLoS One*. 2018;13(5):e0197586.
25. Muthuramalingam K, Singh V, Choi C, Choi SI, Kim YM, Unno T et al. Dietary intervention using (1,3)/(1,6)-β-glucan, a fungus-derived soluble prebiotic ameliorates high-fat diet-induced metabolic distress and alters beneficially the gut microbiota in mice model. *European journal of nutrition*. 2020;59(6):2617–29.
